# In a free healthcare system, why do men not consult for lower urinary tract symptoms (LUTS)?

**DOI:** 10.1186/1447-056X-10-7

**Published:** 2011-06-08

**Authors:** U Chong lai, Yuk Tsan Wun, Tze Chao Luo, Sai Meng Pang

**Affiliations:** 1 Hac Sa Wan Health Centre, Health Bureau, Macau Special Administrative Region, China; 2 Department of Family Medicine and Primary Care, University of Hong Kong, Hong Kong, China

**Keywords:** LUTS, IPSS, help-seeking behaviour, primary care, free healthcare

## Abstract

**Background:**

The prevalence of lower urinary tract symptoms (LUTS) varies among different populations but the rate of seeking medical advice is consistently low. Little is known about the reasons for this low rate. In the city of Macau, China, primary healthcare is free and easily accessible to all citizens. We aim to study the patients' rate of consulting for LUTS and their reasons for not consulting under a free healthcare system.

**Method:**

A convenience sample of 549 male patients aged 40-85 years in a government health centre filled in the International Prostate Symptoms Scale (IPSS) questionnaire. They were also asked if they had consulted doctors for LUTS, and if not, why not.

**Result:**

Of the whole sample, 64 men (11.7%) had ever consulted doctors for LUTS. Of 145 with moderate to severe LUTS, 35 (24.1%) consulted. Of 73 who were dissatisfied with their quality of life, 22 (30.1%) consulted. Regarding the symptoms as normal or not problematic was the main reason for not consulting. Advancing age and duration of symptoms were the significant factors for consulting.

**Conclusion:**

Primary care doctors could help many of LUTS patients by sensitively initiating the discussion when these patients consult for other problems.

## Introduction

Lower urinary tract symptoms (LUTS) are common in men aged 40 years or above [1,2]. The reported prevalence of LUTS varies widely among different age groups and ethnic populations. For men aged 40 or above, the prevalence of moderate to severe LUTS was reported to be: 16.2% (Korea) [3], 19.2% (France) [3], 20.7% (Netherlands) [3], 25.1% (UK) [3], 38% (USA) [4], 56% (Japan) [4]. For men aged 50 or above, the reported rates were: 28% (Denmark) [5], 14% (Singapore) [6], 34% (Malaysia) [6], 39% (Thailand) [6], 41% (UK) [7], 48% (Hong Kong) [6], 59% (Philippines) [6]. These reports used the International Prostate Symptom Score (IPSS) that was validated in different languages to assess the presence and severity of LUTS, and the recruits were from the community-population. Some of them were done concurrently in different countries [3,4,6]. Given that the prevalence of LUTS differs among different populations, more data are required to see if the rates differ even within the same ethnic population.

Though commonly affected, not many men with LUTS consult their doctors for these symptoms. The reported rates vary also widely: 4.4% (US) [8], 9.2% (Denmark) [5], 11.3% (Scotland) [9], 18% (UK) [7], 22.2% (African-American) [10], 38.2% (Spain) [11]. This help-seeking behaviour is known to be affected by the severity of LUTS and how bothered the patients are [7,10,11]. Reports in the literature are mainly on the reasons for men to consult doctors for LUTS [10-12] and not on Asian populations. We are not aware of similar studies on the reasons for not consulting although it was suggested that regarding the symptoms as a normal aging process and embarrassment might deter men from seeking help [13]. Cultural background, accessibility of the healthcare, and financial restraints might affect men's decision to seek medical advice for LUTS. Would more men from a free healthcare system consult doctors for their LUTS?

The Macau Special Administrative Region is a city on the southern coast of China with a population of 549,500 (Year 2010). The government runs seven public outpatient clinics, each on average with 15 doctors, and the service is free to all citizens. Medical consultation is either walk-in or by appointment. Specialist treatment in public hospital is also free to patients aged 65 or above or with low income. In such free and easily accessible setting, we aimed to study (a) the prevalence of LUTS in the Chinese residents of this locality, and (b) how many men with LUTS had sought advice from their doctors and, if not, why not.

## Method

This is a prospective cross-section survey of patients attending the Hac Sa Wan Health Centre, Macau. As other studies we used the IPSS questionnaire to assess LUTS and the effect on the patient's quality of life. In addition we asked whether the patient had ever discussed the symptoms with his doctor, how long had the symptoms been, and the reasons for not seeking help (fear treatment, fear being diagnosed to have prostatic disease, embarrassed to ask, symptoms being normal, or other reasons). To encourage response, the questionnaire was anonymous.

From October 5 to November 11, 2010, a nurse was delegated to explain the aim of the questionnaire to the male patients aged 40 years or above in the waiting room of the adult clinic (after registration and before seeing the doctors). The patients were then invited to fill the questionnaires according to their opinions. The patients who had difficulty with the questionnaire could ask help from the nurse who later collected all the distributed questionnaires. The collection of data stopped when the desired sample size had been reached.

For the estimated sample size (n), we presumed 50% of our recruits would give either a positive or negative answer to the questionnaire items. Taking the confidence level of 95%, margin of error of ± 5%, and p = 0.05 as significant, n is 385. As we anticipated much fewer men in the older group aged 70 or above, we aimed to recruit around 550 men so that the sample in the older group would not be less than 50 or 10% of the total sample. We used chi-squared test, logistic regression and ordinal regression for any significant association or estimation among the categorical data.

The study was approved by the Health Bureau, Macau government.

## Result

In total 549 men completed the questionnaire. The age ranged from 40 to 85 years (mean 57.2 ± 9.38 years). There were 41 men (7.5%) who had none of the LUTS symptoms, giving the prevalence of *any *LUTS of 92.5%. Of the whole sample, 363 (66.1%) were of mild (IPSS score 1-7), 116 (21.1%) of moderate (IPSS score 8-19), and 29 (5.3%) of severe symptoms (IPSS score ≥20). Only 64 men (11.7%) had consulted doctors for LUTS and one of them did not receive any subsequent treatment.

### Age

The severity of LUTS increased with age (Table 1, ordinal regression Wald = 20.41, p < 0.001; the odds ratio for each increase in year being 1.043 (95% confidence interval, CI: 1.024, 1.062)).

**Table 1:** Distribution of age-groups with LUTS severity and consulting doctors

	Age group (years)				Total
		
	40-49	50-59	60-69	≥70	
LUTS severity					

Nil	12 (9.8%)	22 (10.4%)	6 (4.1%)	1 (1.5%)	41 (7.5%)

Mild	84 (68.3%)	144 (67.9%)	97 (66.4%)	38 (55.9%)	363 (66.1%)

Moderate	23 (18.7%)	43 (20.3%)	31 (21.2%)	19 (27.9%)	116 (21.1%)

Severe	4 (3.3%)	3 (1.4%)	12 (8.2%)	10 (14.7%)	29 (5.3%)

Total	123 (100.1%)	212 (100.0%)	146 (99.9%)	68 (100.0%)	549 (100.0%)

Consult doctor					

No	113 (91.9%)	195 (92.0%)	129 (88.4%)	48 (70.6%)	485 (88.3%)

Yes	10 (8.1%)	17 (8.0%)	17 (11.6%)	20 (29.4%)	64 (11.7%)

Total	123 (100.0%)	212 (100.0%)	146 (100.0%)	68 (100.0%)	549 (100.0%)

The older age-group was more likely to consult doctors for their symptoms (Table 1, χ^2 ^= 25.03, p < 0.001). The men who had consulted doctors got LUTS for much more longer time than those who had not (the mean month ± standard deviation were respectively 14.1 ± 16.75 and 4.7 ± 8.87, t-test -4.438, p < 0.001).

### IPSS

Each IPSS item was significantly associated with medical consultation when tested separately (Table 2, all χ^2 ^tests gave p < 0.01). Taking all the items together, logistic regression showed that only nocturia (p = 0.045) and straining (p = 0.007) were significantly associated. The odds ratio of consulting for nocturia was 1.30 (95% CI: 1.005, 1.678), and for straining 1.43 (95% CI: 1.104, 1.845).

**Table 2:** IPSS and whether to consult doctor or not

IPSS	Consult doctor	IPSS score					
		
		0	1	2	3	4	5
Incomplete emptying	No	336	61	38	18	10	22
	
	Yes	27	12	10	5	2	8

Frequency	No	282	84	54	39	9	17
	
	Yes	21	13	13	9	2	6

Intermittency	No	350	53	37	27	8	10
	
	Yes	27	10	7	10	3	7

Urgency	No	372	44	31	21	10	7
	
	Yes	28	11	13	2	4	6

Weak stream	No	368	48	22	24	7	16
	
	Yes	25	9	10	8	5	7

Straining	No	396	37	26	16	4	6
	
	Yes	29	8	10	5	4	8

Nocturia	No	66	221	122	58	11	7
	
	Yes	4	19	17	12	7	5

The men with more severe symptoms were more likely to consult doctors (Table 3, χ^2 ^= 45.10, p < 0.001).

**Table 3:** Association between severity of LUTS and consulting doctor

Consult doctor	LUTS			
	**No LUTS**	**Mild**	**Moderate**	**Severe**

No	39 (95.1%)	336 (92.6%)	94 (81.0%)	16 (55.2%)

Yes	2 (4.9%)	27 (7.4%)	22 (19.0%)	13 (44.8%)

Total	41 (100.0%)	363 (100.0%)	116 (100.0%)	29 (100.0%)

### Quality of life (QoL)

Of the sample, 354 (64.5%) men were in the satisfied range while 73 (13.3%) in the dissatisfied range (Table 4) if the symptoms persisted. Only one man was very dissatisfied; for analysis we grouped this man into the group of "Dissatisfied".

**Table 4:** Association between quality of life and consulting doctor

Consult	Quality of life if symptoms persist						
	
	Very satisfied	Satisfied	Slightly satisfied	Mixed feeling	Slightly dissatisfied	Dissatisfied	Very dissatisfied
No	69 (97.2%)	169 (93.4%)	91 (89.2%)	105 (86.1%)	46 (71.9%)	4 (50.0%)	1 (100.0%)

Yes	2 (2.7%)	12 (6.6%)	11 (10.8%)	17 (13.9%)	18 (28.1%)	4 (50.0%)	0

Total	71 (100.0%)	181 (100.0%)	102 (100.0%)	122 (100.0%)	64 (100.0%)	8 (100.0%)	1 (100.0%)

The probability of consulting doctors increased with dissatisfaction in QoL (logit χ^2 ^= 32.10, p < 0.001); the "slightly dissatisfied" were 13.50 times more likely than the "mixed feeling" to consult a doctor, and the "dissatisfied" were 27.6 times more likely than the "slightly dissatisfied".

When QoL and the total IPSS score were put into the logistic regression model for consulting doctor or not, QoL became insignificant (logit χ^2 ^= 4.19, p = 0.523) while IPSS was still significant (logit χ^2 ^= 16.09, p < 0.001).

### Combined effect

When age, duration of symptoms, severity of symptoms, QoL were put into the logistic regression model, only age and duration were significantly associated with consulting doctors (p = 0.002, p = 0.035 respectively).

### Reasons for not consulting doctors

The most common reason for not consulting doctor was regarding LUTS as normal (Table 5). The "other reasons" given by the patients included: 87 "nothing wrong", 5 "not a problem", 5 "not necessary", 9 "never think of it", 1 "no time", 1 "dislike the idea". The concept of "normal features" had no significant association with severity of LUTS (logit χ^2 ^= 6.41, p = 0.093), QoL ((logit χ^2 ^= 10.74, p = 0.057), or age-group (logit χ^2 ^= 3.07, p = 0.38). Similarly, "feeling embarrassed" was not associated with age-group (logit χ^2 ^= 3.91, p = 0.272).

**Table 5:** Reasons for not consulting doctors for LUTS

Reason	Frequency	
Embarrassed	64	(11.7%*)

Normal features	289	(52.6%)

Fear treatment	22	(4.0%)

Fear diagnosis of prostatic diseases	14	(2.6%)

Others	108	(19.7%)

* Percentage of the total sample

## Discussion

This study on 549 male patients (aged 40 years and above) at a government outpatient clinic in Macau shows a high prevalence of *any *LUTS (IPSS≥1, 92.5%). Most of them (87.2%) were of mild or moderate severity, 13.3% were not satisfied with their quality of life, and only 11.7% consulted their doctors. While age, total IPSS score, quality of life, and duration of symptoms were each significantly associated with consulting doctors, only age and duration were statistically significant if these factors were considered together. The main reason for not consulting was regarding the symptoms as normal or not problematic. Among the IPSS items, nocturia and straining were the important symptoms associated with consulting doctors or not.

The prevalence of IPSS≥1 in our sample is high compared with the 72.3% observed in the study of men aged ≥40 from UK, USA and Sweden [14]. The prevalence of moderate to severe LUTS (IPSS > 7, 145/549 = 26.4%, Table 1) is within the wide range reported by other studies but much lower than those reported by two studies on Chinese. For men aged 50 or above, a study of community residents in Wuhan, Mainland China [15], reported a prevalence of 40.3% for moderate to severe LUTS in 1023 men, and another involving 201 men in Hong Kong was 46% [6]. The equivalent rate in this study was 27.7% (Table 1). It is specially notable that Macau and Hong Kong are two neighbouring cities with over 90% of the population being ethnic Chinese. Although the sampling methods of the three studies were different, the LUTS prevalence might still vary within the same ethnic population.

As regard to men's help-seeking behaviour for LUTS, our findings differ from others in several points. Firstly, nocturia and straining were significantly associated with consulting doctor in this study. The former was the most while the latter the least frequent symptom (Table 2). Many studies found that nocturia was most significant in the association of seeking help [10,16]. This study however found that straining (an obstructive symptom) was more likely than nocturia to associate with seeking help. The statistical significance for nocturia was marginal at p = 0.045.

Secondly, being bothered by LUTS was shown to be significantly associated with consulting doctors [10,11], our study shows that this association was through the severity of LUTS rather than the bother itself. Thus, assessing the bother of LUTS might not be necessary for every population.

Thirdly. it is well known that LUTS is more frequent with advancing age; indeed LUTS incidence was shown to increase linearly with age in one study [17]. But age was seldom reported as an associated factor with seeking medical advice, and one study even showed that age was not associated [10]. In this study, age was the most significant determinant for consulting doctors. The association with symptom severity and bother became insignificant on adjusting for age.

Apart from age, duration of symptoms was the other significant association with consulting doctors. It was likely that men tolerated the symptoms (or tried to "adjust their life to self manage their symptoms" [18]) for some months before they decided to seek treatment. Another explanation was that LUTS started mildly without disturbing daily life. As LUTS progressed with time, and age, the men found them much less tolerable and then consulted their doctors.

Only 11.7% of men in this study consulted their doctors for LUTS. Just 44.8% of men with severe LUTS (Table 3) and 30.1% of men who rated their QoL as dissatisfied (Table 4) consulted their doctors. These rates could be considered low as medical treatment for LUTS was free to our patients. The most common reason for not seeking treatment was taking the symptoms as "normal" or not regarding them as problematic. (Even the "other reasons" for not consulting were in fact regarding LUTS as normal.) The next frequent reason was embarrassment on disclosure. This study supports the speculation that regarding the symptoms as normal and embarrassment deterred men from seeking help [13]. Whether and how much these low rates of help-seeking and patients' perception were due to the Oriental culture need further research.

It is notable in this study that only 30.0% men dissatisfied with their QoL due to LUTS consulted their doctors; the majority were going to tolerate their dissatisfaction some more months. Furthermore, about 10% of those who did not consult were due to perceived embarrassment (Table 5). Primary care doctors could help these patients by initiating sensitively the discussion on LUTS when they consult for other problems.

### Limitations

This study was on a sample from a health centre rather than on a general population. However, the Hac Sa Wan Health Centre serves an area with 39.9% of the Macau population that is typical of the city, and there is likely no large deviation between the study sample and the general population.

Most of our recruits were of mild to moderate LUTS severity; only 5.3% were severe. This might explain the small proportion of patients who were bothered by LUTS and the low rate of seeking medical advice. It is uncertain how much these observation were affected by the sampling method of this study. We took a consecutive convenience sample rather than a randomized one. Due to the busy routines, the nurse did not document the exact number of men who refused the questionnaire but noted from memory to be less than 10%.

## Conclusion

The prevalence of LUTS in ethnic Chinese may vary among different cities. About 10% would seek medical advice even in a free healthcare system. Although the severity of LUTS, the duration of symptoms, the degree of bother and age each had significant association with help-seeking behaviour, only age and duration remain significant in the logistic regression model. The main reason for not seeking help was the perception of the symptoms as being normal aging process. The next important reason was the embarrassment with discussion for which primary care doctors could help with tactful approach.

## Competing interests

The authors declare that they have no competing interests.

## Authors' contributions

All the authors planned the study and read as well as approved the manuscript. Lai UC initiated the study question, supervised the data collection, and helped the preparation of the manuscript. Wun YT analyzed the data, and prepared the manuscript. Luo TC and Pang SM supervised the study and commented the manuscript.
